# Risk Factors for Men’s Lifetime Perpetration of Physical Violence against Intimate Partners: Results from the International Men and Gender Equality Survey (IMAGES) in Eight Countries

**DOI:** 10.1371/journal.pone.0118639

**Published:** 2015-03-03

**Authors:** Paul J. Fleming, Jennifer McCleary-Sills, Matthew Morton, Ruti Levtov, Brian Heilman, Gary Barker

**Affiliations:** 1 Department of Health Behavior, Gillings School of Global Public Health, University of North Carolina, 302 Rosenau Hall, Chapel Hill, NC, 27599-7440, United States of America; 2 Carolina Population Center, University of North Carolina, 211 W. Cameron, Chapel Hill, NC, 27599–7440, United States of America; 3 The World Bank Group, 1818 H Street NW, Washington, DC, 20433, United States of America; 4 Promundo-US, 1367 Connecticut Avenue, NW, Suite #310, Washington, DC, 20036, United States of America; 5 International Center for Research on Women, 1120 20th Street NW, Suite 500 North, Washington, DC, 20036, United States of America; Örebro University, SWEDEN

## Abstract

This paper examines men’s lifetime physical intimate partner violence (IPV) perpetration across eight low- and middle-income countries to better understand key risk factors that interventions can target in order to promote gender equality and reduce IPV. We use data from men (n = 7806) that were collected as part of the International Men and Gender Equality Survey (IMAGES) in Bosnia and Herzegovina, Brazil, Chile, Croatia, Democratic Republic of Congo (DRC), India, Mexico, and Rwanda. Results show that there is wide variation across countries for lifetime self-reported physical violence perpetration (range: 17% in Mexico to 45% in DRC), men’s support for equal roles for men and women, and acceptability of violence against women. Across the sample, 31% of men report having perpetrated physical violence against a partner in their lifetime. In multivariate analyses examining risk factors for men ever perpetrating physical violence against a partner, witnessing parental violence was the strongest risk factor, reinforcing previous research suggesting the inter-generational transmission of violence. Additionally, having been involved in fights not specifically with an intimate partner, permissive attitudes towards violence against women, having inequitable gender attitudes, and older age were associated with a higher likelihood of ever perpetrating physical IPV. In separate analyses for each country, we found different patterns of risk factors in countries with high perpetration compared to countries with low perpetration. Findings are interpreted to identify key knowledge gaps and directions for future research, public policies, evaluation, and programming.

## Introduction

Men’s perpetration of violence against women results from a complex, interconnected ecology of psychological, economic, and sociological factors [1,2]. It is estimated that over 75% of violence against women is perpetrated by their male intimate partners [3]. Intimate partner violence (IPV), also called domestic violence, is defined by the World Health Organization as “any behavior within an intimate relationship that causes physical, psychological or sexual harm to those in the relationship” [4]. Physical IPV, the focus of this paper, includes acts such as punching, kicking, and slapping and is commonly accompanied by psychological (emotional) and sometimes, sexual abuse [5]. The World Health Organization estimates that global prevalence of physical and sexual intimate partner violence (IPV) among ever-partnered women is 30.0%, ranging between 23.2% and 37.7% for different global regions [6]. A meta-analysis conducted on men’s perpetration of IPV (married or cohabiting partner) identifies key characteristics that are correlated with their perpetration of IPV: low marital satisfaction, illicit drug use, and attitudes condoning marital violence [7]. Other important factors included inequitable gender attitudes and depression. Two separate meta-analyses identify witnessing abuse as a child as a moderate risk factor for abuse perpetration in adulthood [8,9].

Additionally, societal factors, such as gender inequalities and patriarchal family structures facilitate a social environment that allows IPV [2]. Settings with unenforced or limited laws preventing violence against women can enable men’s perpetration of IPV [10], and locations of conflict or post-conflict typically have much higher rates of IPV, especially sexual violence [11,12]. These higher rates are partially due to the higher levels of impunity of perpetrators when social institutions that prevent IPV break down or become ineffective, along with increased social and economic stressors on the household [13]. High rates of violence can continue in post-conflict settings if courts and institutions responsible for preventing violence are not established or repaired [14].

Men’s perpetration of IPV is also enabled by prevailing norms related to masculinity and gender equality in most societies [1]. A review of research on the role of masculinity in partner violence presented evidence on different domains of masculinity and male gender norms that influence perpetration [15]. Reviewed articles indicate that men who hold more traditional gender role ideologies (i.e., distinct roles for men and women) are more likely to perpetrate violence [16,17]. Additionally, men who feel stress about their ability to conform to normative ideas regarding what it means to be a man are more likely to perpetrate IPV [18–20]. Societies with greater gender inequities are more likely to teach young men a traditional gender role ideology and increase pressure that men act in traditionally masculine ways, including by perpetrating violence [21].

Power inequalities are central to understanding gender, masculinity and violence perpetration [22,23] and have been the focus of theoretical understandings of masculinity in the past two decades [24–26]. West and Zimmerman [27] conceptualize gender, including masculinity, as constructed through social interactions and put the focus on the actions of individuals. Thus, a man’s masculinity depends on (a) his collection of behaviors and interactions, and (b) how his social environment judges them. Men are often obligated to project a masculine image, often emphasizing strength and power over women. Since power over others is such a critical element of traditionally-defined masculinity, men can sometimes feel a need to assert their power in relationships with women [22]. Because of these normative power dynamics, some men use violence (including physical or other types of violence) to assert power over female partners and thus demonstrate their masculinity [21]. In this way, men’s behaviors, including violence perpetration, help them construct an outward image of power over women that is aligned with a socially constructed ideal of masculinity.

Most international survey research on IPV, women’s rights, and gender equity focuses on women, or has limited data on men’s attitudes and behaviors [3,28]. Research that does focus on risk factors for men’s IPV perpetration has mostly been conducted in high-income countries [7] and it is very limited in low- and middle-income countries. One notable exception is the United Nations (UN) Multi-country Study on Men and Violence in Asia and the Pacific. The UN multi-country study was conducted in six different Asian countries [29]. A recent report from the UN multi-country study examined men’s physical IPV perpetration and found that between 11.5% (rural Indonesia) and 61.9% (Papua New Guinea) of ever-partnered men reported ever perpetrating violence against a partner [29]. The UN multi-country study found that men’s age category, attitudes towards gender equality, childhood experiences of abuse, depression, and history of fighting were all significantly associated with intimate partner violence perpetration. The UN study also analyzed food insecurity, substance abuse (alcohol and illicit drugs) and sexual behaviors and found them to be significantly associated with violence perpetration.

While the UN multi-country study report helps to address a gap in research on men’s perpetration in low- and middle-income countries, it is limited to only six countries in one region of the world. There have been no published studies comparing correlates of men’s lifetime physical IPV perpetration across countries in different continents and regions of the world. Despite the known importance of social and cultural norms for IPV, it is yet to be determined how risk and protective factors for physical IPV perpetration vary in different societies around the world. This paper aims to fill these gaps by analyzing data from eight low- and middle-income countries across five continents to answer three principal research questions: (1) What is the prevalence of self-reported male lifetime perpetration of physical IPV in each setting? (2) What are the main risk and protective factors for physical IPV perpetration across low- and middle-income countries? (3) How do the risk and protective factors for physical IPV perpetration differ among surveyed countries?

## Methods

The data used for these analyses come from the International Men and Gender Equality Study (IMAGES). The IMAGES data collection was conducted between 2009 and 2012 as part of the larger Men and Gender Equality Policy Project co-coordinated by the International Center for Research on Women (ICRW) and Promundo [30–32].

### Settings and Procedures

IMAGES data were collected in eight countries (as of 2012): Bosnia and Herzegovina (Bosnia), Brazil, Chile, Croatia, Democratic Republic of Congo (DRC), India, Mexico, and Rwanda. While sampling strategies varied somewhat by country, IMAGES generally utilized a stratified random sample to select households from which men between ages 18–59 were randomly selected. Data from Bosnia and Rwanda intend to be representative of the entire country, and data from the other countries generally intend to be representative of the regions/cities surveyed. In countries where data collection was not nationally-representative (i.e. Brazil, Chile, Croatia, DRC, India, Mexico), IMAGES sought to follow the World Health Organization multi-country study methodology [3] of collecting data from two urban areas, one major metropolitan area and a secondary city. In general, within a survey location, neighborhoods or blocks were chosen based on population distributions from the most recent census data. Stratified random sampling and probability proportion to size sampling methods were used within each neighborhood or community to ensure the inclusion of adequate sample sizes by age and rural/urban (and socio-economic status in the case of Chile).

The study teams in Chile and Mexico sampled from three metropolitan areas, Croatia from one metropolitan and two rural areas, India from two metropolitan areas, and Brazil from one metropolitan area (See Table 1). The DRC survey intended to understand men’s attitudes and practices in a post-conflict setting and therefore sampled from an urban area (Goma in the North Kivu region), and an internally displaced persons camp, a military base, and two nearby rural villages served as the ‘secondary’ areas. As a result, the DRC sample is unique and, like most of the country samples included, should not be regarded as representative of the country as a whole. Men interviewed men in all locations except for Mexico, where the majority of the surveys with men were conducted by women. Interviewers were specially trained to conduct interviews on sensitive information. Given that we focus on intimate partner violence, we only include men that had ever had a female partner (e.g. ‘ever-partnered men’). The total number of men ages 18–59 interviewed across countries with complete data on our variables of interest was 7806. For more details on the countries included and where the data were collected, see Table 1.

**Table 1:** Details on data collection in each country (adapted from Barker et al. [32] and Levtov [33]).

Data Collection Details	Bosnia	Brazil	Chile	DRC	Croatia	India	Mexico	Rwanda
**Sample size, men**	1532	750	1192	708	1453	1552	1002	2301
**Site(s)**	**Nationally representative sample**	**One metropolitan area:** Rio de Janeiro, two neighborhoods: Maré & Vila Valquiere	**Three metropolitan areas:** Valparaíso, Concepción, & Santiago	**Four areas:** an internally displaced persons camp and a military base, both in Goma and 2 rural villages south of Goma	**One metropolitan area and two rural areas:** Zagreb, & towns and villages in two counties in Eastern Croatia	**Two metropolitan areas:** Delhi & Vijayawada (Tamil Nadu)	**Three metropolitan areas:** Monterrey, Queretaro, & Jalapa	**Nationally representative sample**
**Sample stratification strategy**	Stratified by place of residence	Two income groups: low income (Maré) and middle class (Vila Valquiere), household sample proportional to size of community	Stratified by place of residence and socioeconomic level	Stratified by age and place of residence	Stratified by age and place of residence (rural/urban)	Census block selected by probability proportional to size, systematic random sampling to select household	Stratified by age and place of residence	Stratified by age and place of residence (provinces)
**% of Females in the participating in the labor force, 2010** ^**1**^	34%	59%	47%	71%	46%	29%	44%	86%
**Total Fertility Rate, 2010** ^**1**^	1.2	1.8	1.9	6.3	1.6	2.6	2.3	4.8
**Ratio of females to males in primary and secondary education (%), 2010** ^**1**^	N/A	N/A	1.00	0.78	1.05	0.97	1.03	1.01

^1^Data from World Bank World Development Indicators [77].

While the survey was adapted slightly for each country, each questionnaire had approximately 250 items on men’s attitudes and practices related to daily life, masculinity, employment, health, policies, fatherhood, sexual behaviors, and violence. (see Levtov et al. [33] and Barker et al. [32] for more details on sampling and study design).

### Ethics Statement

Participants verbally consented to participation and interviewers noted this consent on the questionnaires (written consent was not obtained due to low levels of literacy and concerns for respondent’s perception of confidentiality). The study protocol for each country, including informed consent procedures, was approved by the International Center for Research on Women’s institutional review board. Additionally, the protocol was approved by country-specific boards in Chile, Mexico, Rwanda, and the DRC (The Instituto Chileno de Medicina Reproductiva in Chile, The Colegio de Mexico in Mexico, The Rwanda National Ethics Committee in Rwanda, and a technical advisory board in the DRC that was established for this research and was comprised of representatives from the Ministry of Gender, National Institute of Statistics, and the United National Population Fund). In Bosnia, Brazil, Croatia, and India ethical review by local committees was either not available for non-academic research or was not required for non-medical interventions.

### Measures

*Physical violence perpetration against a partner*. Our main dependent variable is self-reported lifetime physical violence perpetration against a partner. The ‘lifetime violence against a partner’ variable is a dichotomous composite variable—if a man reports ever perpetrating at least one instance of five types of physical violence against a partner (1. slapped, 2. pushed, 3. hit with a fist, 4. kicked/dragged/beaten/choked/burned, or 5. threatened or used a weapon), he is coded as having ever perpetrated physical violence against a partner. We based our items and methods on the WHO multi-country study on violence against women [3]. However, given that our study was focused on perpetration and the WHO study focused on women’s victimization, we modified the WHO questions to be about perpetration of any of these forms of physical violence.

*Risk and protective factors*. Based on available data and the previous literature, our main risk/protective factor variables were: (1) having permissive attitudes towards violence against women (VAW), (2) the Gender Equitable Men (GEM) scale, (3) witnessing of inter-parental violence, (4) having been involved in fights with a weapon, and (5) experiencing depression.

Permissive attitudes towards VAW were assessed by whether or not the man ‘strongly agreed’ or ‘partially agreed’ with the statement: ‘there are times when a woman deserves to be beaten.’

The GEM scale measures the extent to which men agree with gender equality or separate roles for men and women, and has demonstrated satisfactory validity and reliability in previous psychometric testing [34,35] as well as with the current samples [33]. GEM scale example items include: “A man should have the final word about decisions in his home,” “Changing diapers, giving kids a bath, and feeding the kids are the mother’s responsibility,” and “To be a man, you need to be tough.” The GEM scale items included in the survey were slightly different in each country for cultural relevance (i.e. local study staff occasionally recommended adding or subtracting an item based on the local context). Final country-specific scales used in our analyses were developed using factor analysis to select the appropriate items [33]. Using these final items selected in the factor analysis for each country, each man’s GEM score was created as an additive scales where higher scores indicate more equitable attitudes [33]. The final number of items and Cronbach’s α for each country were: Bosnia (15 items, α = 0.85), Brazil (11 items, α = 0.89), Chile (15 items, α = 0.67), Croatia (13 items, α = 0.83), DRC (13 items, α = 0.76), India (12 items, α = 0.75), Mexico (11 items, α = 0.70), Rwanda (13 items, α = 0.99) [33]. For descriptive results, to compare across countries with different number of items, we scaled men’s scores across countries to equal possible ranges (scores range between 0 and 2; 0 = most gender inequitable and 2 = most gender equitable). For the multivariate analyses, we used a standardized GEM score based on the mean and standard deviation for each country. A higher standardized GEM scale score indicates more supportive attitudes towards gender equity compared to other participants from the same country. For specific items included in each country’s GEM scale, see Levtov et al. [33].

Witnessing of inter-parental violence was measured by a survey item stating, “I saw my mother being beaten by her husband or boyfriend.” Men who responded “sometimes,” “often,” or “very often,” were coded as a “witness of intra-parental violence.”

To measure involvement in fighting or violence generally, we used a fighting variable that measured whether or not the man had ever been involved in a fight with a knife or other weapon. This question was not specific to intimate partners, but rather asked generally. There was no available variable about fighting without weapons.

For the depression variable, men were asked how often they felt “depressed” in the past month and those who responded “often” or “sometimes” in the past month were coded as a “1” (i.e. “depressed”) for the dichotomous depression variable. This is not a clinical measure of depression, but rather a subjective self-report of having experienced depression or not. This item was not asked in the DRC or Rwanda.

*Demographic variables*. We analyzed various demographic variables (shown in previous research to be associated with IPV), including: age, education, marital/cohabitation status, employment status, and income. Age is divided into three categories (18–29, 30–39, 40–59) and education is also divided into three categories (no education or primary school, secondary schooling, post-secondary school). Marital/cohabitation status was measured dichotomously (1 = married or cohabiting, 0 = not married or cohabiting). The employment variable was measured dichotomously (1 = formal/informal work, 0 = never worked, unemployed, or retired). We created a four-category income variable that captured relative income within each country: low income quartile, mid-low income quartile, mid-high income quartile, high-income quartile (see Levtov[33]). This variable was intended to create income quartiles for each country. Each quartile had roughly 25% of participants, but this was not always possible for countries like the DRC where income was asked as a categorical question. This variable should be interpreted as a rough division of income within each country’s sample. It is possible, for example, that those in the highest income quartile in our analyses are actually in a lower income quartile relative to the entire country population.

### Data Analysis

We present descriptive and logistic regression analyses in this paper. For descriptive tables, we used frequencies by country and the all-country sample to describe participants’ demographics, attitudes and behaviors (Table 2). We also examined bivariate relationships between physical violence perpetration and key demographic, attitudinal, and behavioral variables for each country and overall, using t-tests for continuous variables and χ^2^ tests for dichotomous variables. We use p value <0.05 as our criteria for a statistically significant result, however we still highlight results that are marginally non-significant (p<0.10) as potentially meaningful to understanding risk factors for violence perpetration.

**Table 2:** Demographic and other characteristics of men in the International Men and Gender Equality Survey (IMAGES).

		Bosnia		Brazil		Chile		Croatia		DRC		India		Mexico		Rwanda		All-Country	
		*n* = 1169		*n* = 617		*n* = 1051		*n* = 1152		*n* = 539		*n* = 917		*n* = 895		*n* = 1456		*n* = 7806	
**Age**		n	%	n	%	n	%	n	%	n	%	n	%	n	%	n	%	n	%
*18–28*		525	44.9	229	37.1	352	33.5	407	35.3	111	20.6	183	20.0	323	36.1	176	12.1	2309	29.6
*29–39*		346	29.6	156	25.3	262	24.9	344	29.9	207	38.4	404	44.1	238	26.6	539	37.0	2499	32.0
*40–59*		298	25.5	232	37.6	437	41.6	401	34.8	221	41.0	330	36.0	334	37.3	741	50.9	2998	38.4
**Education**																			
*No Formal Education*		1	0.1	15	2.4	11	1.1	0	0.0	72	13.4	133	14.5	11	1.2	244	16.8	487	6.2
*Up to Primary School*		42	3.6	326	52.8	105	10.0	33	2.9	157	29.1	93	10.1	101	11.3	983	67.5	1840	23.6
*Secondary School*		760	65.0	201	32.6	501	47.7	701	60.9	209	38.8	340	37.1	218	24.4	168	11.5	3103	39.8
*Post-Secondary School*		366	31.3	75	12.2	434	41.3	418	36.3	101	18.7	351	38.3	565	63.1	61	4.2	2376	30.4
**Marital/Residential Status**																			
*Married and/or cohabitating*		480	46.8	342	77.4	462	44.0	568	67.7	432	80.9	812	89.2	382	53.2	1317	90.5	4795	61.5
**Employment Status**																			
*Currently employed*		713	61.0	468	75.9	758	72.1	830	72.1	359	66.6	878	95.8	715	79.9	1425	97.9	6154	78.8
**Risk Factors for Violence Perpetration**																			
*Permissive attitudes towards VAW*		244	20.9	123	19.9	106	10.1	126	10.9	332	61.6	591	64.5	51	5.7	268	18.4	1841	23.6
*Witness of Intra-parental violence*		117	10.0	96	15.6	331	31.5	178	15.5	236	43.8	391	42.6	158	17.7	650	44.6	2158	27.7
*Average GEM Score response*	*mean*	1169	1.4	617	1.4	1051	1.5	1152	1.7	539	0.9	917	0.9	895	1.6	1456	1.1	7806	2.3
	*(SD)*		(0.4)		(0.5)		(0.3)		(0.3)		(0.4)		(0.4)		(0.4)		(0.4)		(0.5)
*Ever fought w/ a weapon*		223	19.1	139	22.5	164	15.6	210	18.2	66	12.2	60	6.5	103	11.5	74	5.1	1040	13.3
*Depressed in last month*		275	23.5	47	7.6	172	16.4	367	31.9	NA	NA	242	26.4	81	9.1	NA	NA	NA	NA
**Violence Perpetration**																			
*Ever slapped a partner*		181	15.5	77	12.5	199	19.1	224	19.5	203	38.1	257	28.0	116	13.0	480	33.0	1738	22.3
*Ever pushed a partner*		219	18.8	125	20.3	255	24.5	304	26.5	187	35.4	194	21.2	75	8.4	327	22.5	1689	21.6
*Ever hit a partners with fist*		70	6.0	15	2.4	60	5.7	56	4.9	123	23.0	84	9.2	20	2.2	154	10.6	582	7.5
*Ever kicked/dragged/beaten a partner*		26	2.2	55	8.9	12	1.2	33	2.9	72	13.4	68	7.4	8	0.9	47	3.2	321	4.1
*Ever used a weapon w/ partner*		33	2.8	8	1.3	17	1.6	28	2.4	23	4.3	15	1.6	15	1.7	14	1.0	153	2.0
*Lifetime violence against a partner*		283	24.2	152	24.6	308	29.3	366	31.7	243	45.1	342	37.3	152	17.0	569	39.1	2418	31.0

We conducted multivariate logistic regression analyses to examine the influence of selected variables on physical violence perpetration. First, we ran a logistic regression model separately for each country. Then, we conducted the analysis with all eight countries simultaneously using country fixed effects to examine the relationship between perpetration and the independent variables across all countries. For the all-country model, we excluded variables that were not asked in all eight countries (e.g., depression). Observations with missing data on any of the included variables were excluded from the analyses (i.e., listwise deletion). All analyses were conducted using SAS version 9.3.

## Results

Of the 7,806 men in our analytic sample, 2415 (30.9%) report ever having perpetrated physical violence against a partner. The share of men who reported lifetime perpetration of physical violence was 17% in Mexico, 24% in Bosnia, 25% in Brazil, 29% in Chile, 32% in Croatia, 37% in India, 39% in Rwanda, and 45% in DRC. Demographic, attitudinal, and behavioral information for men in our sample ranged greatly for each country and is presented in Table 1.

### Comparing Perpetrators and Non-Perpetrators

We conducted simple logistic regression analyses between violence perpetration and key demographic and behavioral variables (See Table 3 for multi-country analysis and Table 4 for individual country analyses). Results from the multivariate logistic regression with the overall eight-country sample with country fixed effects show that older age, witnessing intra-parental violence, permissive attitudes towards VAW, lower GEM score, and being involved in fights were all significant risk factors for having self-reported perpetrating physical IPV against a partner (see Table 3). When controlling for other variables in the model, the odds of reporting ever having reported perpetrating physical IPV for men between ages 40–59 were nearly twice the odds of men between ages 18–28 (Adjusted Odds Ratio (AOR): 1.88, 95% CI: 1.47–2.41). Men who held permissive attitudes towards VAW had nearly twice the odds of perpetrating IPV (AOR: 1.70, 95% CI: 1.34–2.16). Men who witnessed their mother being beaten by a partner had more than 2.5 times the odds of ever having perpetrated violence against their own partners (AOR: 2.53, 95% CI: 2.08–3.07). Men’s GEM scores were also significantly correlated with violence perpetration. For every one standard deviation increase in men’s GEM score (indicating greater support for gender equality relative to other men in his country sample), men had more than 10% lower odds of perpetrating violence against a partner (AOR: 0.89, 95% CI: 0.80–0.97). Finally, men who had been involved in at least one fight with a weapon had greater odds of having perpetrated physical IPV compared to those men who had not been in a fight with a weapon (AOR: 2.38, 95% CI: 1.91–2.97). When controlling for all the other variables in the model, income quartile, employment status and level of education were not significantly associated with reporting perpetration of physical violence against a partner.

**Table 3:** Results from multivariate logistic regression, correlates of lifetime physical violence perpetration against a partner, presented as unadjusted and adjusted odds ratios^a^ (all-country sample, n = 7806).

Demographic, Attitudinal, and Behavioral Variables	Frequency (%) or Mean	OR (n = 7810)	95% CI	AOR^a^ (n = 7810)	95% CI
*Age 18–28 (REF)*	29.6	1.00	--	1.00	--
*Age 29–39*	32.0	1.69*	1.41–2.02	1.56**	1.35–1.80
*Age 40–59*	38.4	2.04*	1.68–2.48	1.88**	1.47–2.41
*No Schooling or Primary (REF)*	29.8	1.00	--	1.00	--
*Secondary School*	39.8	0.78*	0.63–0.96	0.95	0.75–1.21
*Post-Secondary School*	30.4	0.55*	0.43–0.70	0.76	0.55–1.04
*Low Income (REF)*	24.4	1.00		1.00	--
*Mid-Low Income*	30.6	1.13	0.88–1.45	1.11	0.93–1.33
*Mid-High Income*	26.4	1.15	0.97–1.36	1.17**	1.04–1.31
*Highest Income*	18.5	0.93	0.78–1.12	0.96	0.75–1.22
*Employed*	78.8	1.36*	1.13–1.64	1.08	0.94–1.23
*Permissive attitudes towards VAW*	23.6	2.32*	1.67–3.22	1.70**	1.34–2.16
*Witness of Intra-parental violence*	27.7	3.09*	2.50–3.83	2.53**	2.08–3.07
*GEM Score* ^*b*^ *(mean)*	1.3	0.75*	0.67–0.84	0.89*	0.80–0.97
*Has been involved in Fights*	13.3	2.30*	1.75–3.02	2.38**	1.91–2.97

* p<.05,

**p<.01; CI = Confidence Intervals, OR = unadjusted odds ratio; AOR = Odds ratios adjusted for other variables in table

^a^Adjusted for all other variables presented in table

^b^ The GEM score variable used in this regression model is standardized within each country with a mean of 0 and a standard deviation of 1. Thus, for the regression model, the GEM score variable measures your GEM score relative to other men surveyed in the same country. The mean reported in this table is the mean for man’s average GEM score. In both cases, the higher the number, the more gender equitable.

**Table 4:** Results from multivariate logistic regression, correlates of lifetime physical violence perpetration against a partner, presented as unadjusted odds ratios and adjusted odds ratios^a^.

Demographic, Attitudinal, and Behavioral Variables	Bosnia (n = 1169)				Brazil (n = 617)			
	OR	95% CI	AOR	95% CI	OR	95% CI	AOR	95% CI
*Age 18–28 (REF)*	.00		1.00	*-*	.00	-	1.00	
*Age 29–39*	.08	0.78–1.48	1.22	0.84–1.76	.01	0.62–1.63	1.27	0.73–2.20
*Age 40–59*	.27	0.92–1.77	1.24	0.83–1.83	.16	0.76–1.76	1.31	0.79–2.18
*No Schooling or Primary School (REF)*	.00	-	1.00	*-*	.00	-	1.00	
*Secondary School*	.69	0.36–1.33	0.79	0.39–1.60	.76	0.51–1.13	0.76	0.47–1.22
*Post-Secondary School*	.57	0.29–1.13	0.72	0.34–1.55	.26**	0.11–0.58	0.47	0.19–1.13
*Low income quartile (REF)*	.00	-	1.00	*-*	.00	-	1.00	
*Mid-Low Income quartile*	.82	0.56–1.19	0.82	0.53–1.26	.01	0.55–1.86	0.98	0.42–2.29
*Mid-High Income quartile*	.91	0.59–1.42	1.06	0.63–1.78	.91	0.53–1.54	1.00	0.44–2.27
*Highest Income quartile*	.80	0.45–1.44	0.91	0.47–1.80	.59	0.32–1.08	0.64	0.26–1.59
*Employed*	.97	0.74–1.27	1.08	0.75–1.56	.03	0.67–1.59	1.15	0.58–2.26
*Permissive attitudes towards VAW*	.75**	2.03–3.72	1.34	0.93–1.95	.40**	1.57–3.66	1.92**	1.18–3.12
*Witness of Intra-parental violence*	.07**	2.75–6.03	2.77**	1.80–4.26	.10**	1.32–3.33	1.71*	1.03–2.85
*GEM Score (standardized)*	.58**	0.50–0.66	0.68**	0.58–0.80	.81*	0.68–0.97	0.99	0.79–1.23
*Has been involved in Fights*	.56**	2.62–4.85	2.92**	2.09–4.08	.90**	3.26–7.37	4.04**	2.62–6.23
*Depressed*	.50**	1.11–2.03	1.14	0.81–1.60	.98**	1.63–5.46	2.44**	1.26–4.73

Demographic, Attitudinal, and Behavioral Variables	Chile (n = 1051)				Croatia (n = 1152)			
	OR	95% CI	AOR	95% CI	OR	95% CI	AOR	95% CI
*Age 18–28 (REF)*	.00		1.00		.00		1.00	
*Age 29–39*	.59*	1.11–2.30	1.47	0.95–2.26	.66**	1.20–2.31	1.66**	1.14–2.41
*Age 40–59*	.01**	1.46–2.77	1.78**	1.18–2.69	.86**	2.10–3.90	2.42**	1.70–3.44
*No Schooling or Primary School (REF)*	.00	-	1.00		.00	-	1.00	
*Secondary School*	.58*	0.38–0.88	0.92	0.58–1.49	.99	0.47–2.08	1.45	0.60–3.51
*Post-Secondary School*	.51**	0.33–0.77	1.06	0.63–1.79	.83	0.39–1.77	1.25	0.50–3.11
*Low income quartile (REF)*	.00	-	1.00		.00	-	1.00	
*Mid-Low Income quartile*	.31	0.90–1.89	0.94	0.58–1.51	.46*	1.05–2.04	1.19	0.79–1.79
*Mid-High Income quartile*	.24	0.84–1.83	1.07	0.64–1.77	.54*	1.07–2.22	1.35	0.86–2.13
*Highest Income quartile*	.07	0.65–1.78	0.90	0.48–1.69	.09	0.74–1.60	0.98	0.60–1.60
*Employed*	.57**	1.15–2.15	1.44	0.95–2.20	.21	0.91–1.60	1.03	0.72–1.48
*Permissive attitudes towards VAW*	.59**	1.73–3.89	1.90**	1.18–3.04	.50**	3.05–6.63	3.14**	2.00–4.95
*Witness of Intra-parental violence*	.20**	2.42–4.23	2.68**	1.99–3.60	.97**	2.14–4.11	2.10**	1.47–3.00
*GEM Score (standardized)*	.74**	0.65–0.84	0.87	0.74–1.01	.68**	0.60–0.77	0.87	0.75–1.02
*Has been involved in Fights*	.75**	1.96–3.87	2.91**	1.98–4.27	.43**	1.79–3.29	1.98**	1.41–2.76
*Depressed*	.64**	1.17–2.31	1.50*	1.02–2.21	.55**	1.20–2.02	1.29	0.97–1.72

Demographic, Attitudinal, and Behavioral Variables	DRC (n = 539)				India (n = 917)			
	OR	95% CI	AOR	95% CI	OR	95% CI	AOR	95% CI
*Age 18–28 (REF)*	.00		1.00	*-*	.00	-	1.00	
*Age 29–39*	.25**	1.36–3.70	2.47**	1.45–4.22	.80**	1.22–2.65	1.93**	1.26–2.95
*Age 40–59*	.15**	1.92–5.17	3.91**	2.29–6.69	.31**	1.55–3.44	2.57**	1.65–3.99
*No Schooling or Primary School (REF)*	.00	-	1.00	*-*	.00	-	1.00	
*Secondary School*	.71**	1.17–2.51	1.90**	1.26–2.87	.70*	0.50–0.99	0.68	0.46–1.01
*Post-Secondary School*	.40	0.87–2.24	2.02*	1.14–3.56	.49**	0.35–0.69	0.47**	0.30–0.74
*Low income quartile (REF)*	.00	-	1.00	*-*	.00	-	1.00	
*Mid-Low Income quartile*	.98**	1.51–5.87	2.80**	1.36–5.79	.20	0.84–1.73	1.42	0.94–2.15
*Mid-High Income quartile*	.80	0.91–3.57	1.17	0.56–2.47	.98	0.67–1.43	1.34	0.84–2.14
*Highest Income quartile*	.17	0.45–3.01	0.76	0.26–2.17	.91	0.62–1.33	1.35	0.82–2.24
*Employed*	.32	0.92–1.90	1.10	0.71–1.71	.06	0.55–2.08	1.41	0.65–3.05
*Permissive attitudes towards VAW*	.86**	1.31–2.66	1.52	0.99–2.34	.47**	1.11–1.96	1.28	0.89–1.84
*Witness of Intra-parental violence*	.26	0.90–1.78	1.26	0.86–1.83	.09**	3.08–5.42	3.73**	2.77–5.04
*GEM Score (standardized)*	.80**	0.67–0.95	0.92	0.75–1.14	.88	0.77–1.02	1.03	0.85–1.24
*Has been involved in Fights*	.17	0.70–1.96	1.32	0.75–2.33	.39**	1.96–5.88	3.51**	1.92–6.43
*Depressed*	NA	NA—NA	NA	NA	.67**	1.24–2.25	1.65**	1.18–2.32

Demographic, Attitudinal, and Behavioral Variables	Mexico (n = 895)				Rwanda (n = 1456)			
	OR	95% CI	AOR	95% CI	OR	95% CI	AOR	95% CI
*Age 18–28 (REF)*	.00		1.00		.00		1.00	
*Age 29–39*	.52	0.96–2.40	1.29	0.78–2.16	.40	0.98–2.01	1.43	0.99–2.08
*Age 40–59*	.54*	1.01–2.35	1.18	0.72–1.93	.46*	1.03–2.07	1.55*	1.08–2.22
*No Schooling or Primary School (REF)*	.00	-	1.00		.00	-	1.00	
*Secondary School*	.27	0.71–2.26	1.48	0.77–2.81	.18	0.85–1.63	1.04	0.73–1.48
*Post-Secondary School*	.82	0.48–1.39	1.40	0.75–2.61	.71	0.41–1.23	0.68	0.38–1.21
*Low income quartile (REF)*	.00	-	1.00		.00	-	1.00	
*Mid-Low Income quartile*	.86	0.45–1.66	0.95	0.47–1.93	.95	0.71–1.28	1.01	0.74–1.37
*Mid-High Income quartile*	.29	0.69–2.39	1.64	0.83–3.23	.15	0.85–1.54	1.17	0.86–1.59
*Highest Income quartile*	.87	0.46–1.64	1.19	0.59–2.42	.08	0.79–1.49	0.92	0.76–1.49
*Employed*	.70*	1.04–2.78	1.87	0.98–3.54	.02	0.49–2.11	0.82	0.35–1.94
*Permissive attitudes towards VAW*	.55**	2.54–8.15	2.55**	1.31–4.97	.55**	1.19–2.02	1.33	0.99–1.80
*Witness of Intra-parental violence*	.81**	2.58–5.63	3.08**	2.00–4.72	.42**	1.95–3.00	2.34**	1.88–2.92
*GEM Score (standardized)*	.61**	0.52–0.72	0.68**	0.56–0.82	.88*	0.79–0.98	0.94	0.83–1.06
*Has been involved in Fights*	.67**	1.69–4.21	2.19**	1.31–3.66	.69*	1.06–2.70	1.67*	1.02–2.73
*Depressed*	.42**	1.45–4.01	1.85*	1.06–3.23	A	NA	NA	NA

* p<.05,

**p<.01;

CI = Confidence Intervals, OR = unadjusted odds ratio; AOR = Odds ratios adjusted for other variables in table;

^a^Adjusted for all other variables presented in table.

Table 4 shows the results from the individual country analyses. While many of the relationships from the pooled sample were consistent in individual country analyses, we find substantial variation in the significance, direction, and strength of associations across countries. Age effects were significant only in Chile, Croatia, DRC, India, and Rwanda. Notably, education effects were significant only in DRC and India, the countries with the first highest and third highest prevalence of violence perpetration. But they operate in opposite directions in the two countries (DRC men with higher education are *more* likely to perpetrate violence and Indian men with higher education are *less* likely to perpetrate) and are therefore difficult to interpret. Permissive attitudes towards VAW were not significant risk factors for violence perpetration in Bosnia and India (and the AORs were marginally non-significant for the DRC and Rwanda sample). Witnessing one’s own mother being abused was significantly associated with IPV perpetration everywhere except for the DRC. GEM score was significantly associated with IPV perpetration in Bosnia and Mexico, marginally non-significant in Chile and Croatia, and not significant in the other countries. Having fought with a weapon was one of the most consistent correlates of IPV perpetration; it was significant everywhere except for the DRC. While we could not include depression as an indicator in the all-country model because the question was not asked in two countries (Rwanda and DRC), it was a risk factor for physical IPV perpetration in all applicable countries (it was only marginally non-significant in the Bosnia and Croatia multivariate analyses).

## Discussion

A quarter or more of participants in most of the countries had ever perpetrated physical violence against a partner. The estimates from this analysis are similar to those found in the 2013 WHO study related to IPV prevalence [6] and from the recent UN Multi-country study [29]. This analysis pointed to several modifiable risk and protective factors that may be able to decrease violence perpetration by men.

We found that, among those variables measured, the factors most strongly associated with self-reported physical IPV perpetration were witnessing intra-parental violence and having been involved in fights with a weapon—both were highly significant in the all-country sample and statistically significant in nearly every country. Having permissive attitudes towards VAW, an inequitable GEM score, and older age were also significant risk factors in the all-country sample and some of the individual country samples. Reporting depression was significantly correlated with physical IPV perpetration in the countries where this was asked.

The strength and significance of the correlation between witnessing of inter-parental violence and perpetrating physical IPV suggests evidence of the intergenerational transmission of behaviors and gender norms. This supports previous evidence highlighting the importance of witnessing violence as a child for men’s future aggression against women and reinforces its generalizability to a range of developing country contexts [17,36,37]. A meta-analytic review of 39 published research studies on the intergenerational transmission of partner violence demonstrated that children who witness parental violence are themselves more likely to be involved in violent relationships in adulthood [8]. This increased likelihood of violence among those who witness violence is in part driven by psychosocial concepts from the Social Learning Theory [38] and its subsequent version, Social Cognitive Theory [39]. These behavioral theories have established that individuals learn how to behave socially through observing and imitating important others in their social environment. This observation and imitation occurs throughout the lifespan, but can be particular important for children and youth. Interrupting this cycle is critical to reducing violence perpetration. Additionally, though our study did not examine experiences of child abuse, it often co-occurs with intimate partner violence and may also contribute to this cycle of violence and should be addressed in violence prevention efforts.

Reports of getting into fights was also an important factor associated with physical IPV perpetration for men in most but not all countries. This may be connected to the finding that men who are depressed were more likely to perpetrate physical IPV since they both relate to how men learn to express their emotions and anger. For example, a man suffering from depression may take out feelings of sadness and loneliness by using violence against a partner [3,40]. Conversely, this finding could demonstrate the negative mental health effects of perpetration on men. In either case, efforts to improve mental health for men may help reduce IPV perpetration. The finding related to fighting also highlights the fact that violence between men is linked to violence against women. Kaufman [41] refers to the interrelatedness of types of violence as men’s ‘triad of violence’, including violence against women, violence against other men, and violence against self. Efforts to prevent IPV may also need to address men’s three types of violence perpetration and the masculine norms that support all forms. The findings imply that policy efforts which take a narrow view of specific types of violence may miss opportunities to address interconnected violent attitudes and behaviors more holistically.

Men with more inequitable gender attitudes—and those with more permissive attitudes towards violence against women—were more likely to have perpetrated IPV. The masculine norm in many societies is often characterized by being aggressive, unemotional, and dominant over women. Men who are supportive of these traditional norms and accepting of men’s violence against women may be more concerned with how others perceive them. Men often feel culturally compelled to project a masculine image; the potential consequences for individual men who are perceived as non-masculine can include social ostracization [42] or even death by violence [43]. In this way, men’s behaviors, including violent behaviors, are linked to their projection of a masculine identity for their family, community, and peers [21]. Men can use violence against female partners as a way to demonstrate their masculinity and assert their dominance [15,20], especially in contexts where community norms are also supportive of violence and inequitable norms [44]. Additionally, like witnessing violence, there is evidence that attitudes towards violence against women, gender roles, and equality are also passed between generations [32,45]. Changing men’s attitudes would likely influence their own children to be more equitable as well [31].

We found that younger (ever-partnered) men were less likely to have perpetrated violence against a partner. Previous studies of the impact of age on lifetime physical violence perpetration have had mixed results [40,46]. It is possible that our findings simply indicate that younger men have had fewer opportunities to perpetrate. Previous analyses with IMAGES data have shown that older men tend to have more permissive attitudes towards violence against women. This may represent an additional explanation of the higher prevalence of perpetration among older men [47]. Thus, it is possible that younger men’s perpetration, and attitudes, represent societal shifts in acceptability of violence against women. Future survey research should examine this question longitudinally and measure IPV exposure of the last 12 months as well as lifetime prevalence, as recommended by recent UN guidance on minimum gender indicators [48].

Though education level was non-significant in the all-country sample, we found some limited evidence in the country samples that increased schooling may have a protective effect where those with more schooling were less likely to perpetrate violence than those with less schooling. But, the opposite was true in the DRC and there was a non-significant relationship in most countries when controlling for other factors. The findings in the DRC may be due to the unique sample which was drawn in part from a military base and internally displaced persons camp. Previous evidence has shown that education for women and girls can have a positive effect on their communities and their own agency [49]; the role of men’s education in creating gender equitable attitudes and communities and preventing violence is less clear. Further complicating the issue, there is some evidence that the interaction between men’s and women’s education can be a risk factor for violence[50]. Our multivariate results can be contrasted with previous studies examining the effect of men’s education on men’s violence that show that greater levels of men’s education are associated with less perpetration [51–53]. Previous IMAGES analysis, and our own analyses, showed that increased education was associated with more gender-equitable attitudes which in turn is a protective factor for violence perpetration [32,33,54]. Given that GEM score and attitudes towards VAW were also included in our model, the relationship between education and violence perpetration may be attenuated by other factors in our multivariate analyses.

While some risk factors were important in almost every country, countries with high violence perpetration such as DRC and India had slightly different patterns of risk factors than countries with lower perpetration. Pierotti argues that as communities and countries are increasingly exposed to global cultural scripts that are opposed to VAW, they adopt attitudes in opposition to VAW [55]. Countries that have increased engagement with the global economy may create opportunities for their citizens to be more exposed to “global cultural scripts” [55]. Risk factors for IPV perpetration may be different in countries or settings where global cultural scripts in opposition to VAW have not been entrenched (as evidenced in our sample by high prevalence of permissive attitudes towards VAW in DRC and India). In those settings, there may be other factors that were not in our model that are contributing to violence perpetration. Future research with men could explore the extent to which risk factors, including permissive attitudes toward violence, vary by the prevalence of perpetration in a country.

### Limitations

The IMAGES dataset is a rich source of information on men’s attitudes and practices across the globe, but it has limitations. Because these data are cross-sectional, we are unable to make claims of the sequencing of events or causality. For example, while having more equitable gender attitudes could result in decreased use of violence, other explanations could exist. For instance, attitudes could be established after perpetration or other unmeasured factors could underlie the relationship. In order to establish causality, longitudinal designs would be needed in the future. Another limitation is that, with the exception of Bosnia and Rwanda, the samples are not nationally representative. As such, the findings are limited to the region or location where the data were collected.

Because survey instruments varied slightly and were administered in each country’s language, there were certain variables that may have carried slightly different meanings in each location (despite double-back translation), potentially limiting comparability across countries. Self-reported measures used across a variety of settings could also result in variation in how participants responded and thus could introduce bias. Additionally, there may be some factors that were not measured or not included in the models that could have changed the results of our models.

There are several potential limitations due to our measurement of lifetime violence perpetration. First, we only focused on physical violence perpetration, not other types of violence such as financial, emotional, or sexual violence. Second, our measure—though aligned with standard measures of physical intimate partner violence—asked about specific types of physical violence and may have missed other types of physical violence not included in the questions. Further, one question asked about ‘use’ or ‘threats’ and thus could capture men who *threatened* to use a gun or other weapon but did not actually perpetrate physical violence. Third, men’s responses could have been inaccurate due to recall bias, social desirability bias, or concerns about being prosecuted for their violence. Previous studies have noted concerns related to potential underreporting of men’s self-reported violence perpetration [56–59]. Social desirability bias is a particular concern in Mexico where men were interviewed by women. Nonetheless, if there was underreporting of IPV, it was generally consistent with that of women: the IMAGES survey additionally interviewed a sample of women (not necessarily partners of men surveyed) and found that in every country except for Mexico and Bosnia, the proportion of women reporting victimization was approximately equal to the proportion of men reporting perpetration. In Bosnia and Mexico, the percentage of women reporting victimization was approximately 15 points higher than the percentage of men reporting perpetration which could cause bias in the analyses from those countries (i.e. some men categorized as ‘non-perpetrators’ in our analyses could be perpetrators).

### Future Directions for Research, Policy and Practice

Expanding efforts such as the IMAGES and UN Multi-country Study to more countries will help establish the foundation for interventions, policies and future research on violence perpetration, gender, and masculinity. Additionally, conducting regular nationally-representative surveys with men over time would allow for tracking shifts in attitudes and behaviors related to gender equality, including the reduction of VAW [60].

The findings contribute further evidence suggesting that changing men’s attitudes and perpetration behaviors could influence their own children to be more equitable—and less violent—as well [31]. A 2007 World Health Organization, as well as a recent systematic review, provides evidence that gender-transformative interventions, which seek to change men’s conceptualization of gender norms, can be effective in reducing violence perpetration and changing harmful attitudes and behaviors [61,62]. While still very limited in terms of rigorous evaluation, much of the existing intervention research indicates that men critically discussing gender norms in groups, often combined with community campaigns to promote norm change, can help to start break down some of the harmful norms and attitudes associated with traditional masculine norms [61–66]. Using mass media to challenge violence and transform gender norms has been another useful strategy [67–69]. Changing the context and cultural scripts around masculinity through mass media can create an environment that is supportive of a shift in men’s behaviors and attitudes.

There is a pressing need for large-scale evaluations of programs and policies that target men to reduce perpetration of violence, increase support for gender equality, and engage men in active fatherhood. For example, modifications to school curricula can help challenge harmful norms of masculinity that promote violence perpetration [70,71] and evaluations of these curricula can help diffuse these changes to other settings. Policies and programs related to pre-natal, post-natal, and child health that are more inclusive of fathers have the potential to promote men’s role as a supportive, care-giver from the start of their child’s life [30]. Evaluations of such policies and programs are needed to understand whether they break down the social norms around gendered division of labor at the heart of gender equality [31]. Finally, given the strong association between having witnessed IPV in home of origin and later use—and the finding from the WHO multi-country studies that women who witness IPV against their mothers growing up are more likely to be victims of IPV as adults [3]—the issue of how to break this transmission must be central to prevention efforts. Psychosocial support models that provide specific assistance to those who are exposed to violence have been underutilized and could help break this cycle [72].

To date, most of the evaluations of these types of gender-transformative interventions and programs have been small in scale and involved short follow up periods [62,64,65,73], though there are a few notable exceptions [74–76]. Future research should prioritize rigorous experimental and quasi-experimental evaluations, including at the community and societal levels, to better understand the extent of effectiveness and generalizability—as well as key elements—of programs that target men and boys to reduce violence and change gender dynamics.

## Conclusion

Men’s perpetration of violence against women is intimately interrelated with gender norms and gender inequalities. Intimate partner violence, and other forms of violence against women, are important factors inhibiting women’s agency and preventing greater gender equality. Reducing violence against women can result in greater gender equality; this increased gender equality, in turn, is essential to greater achievement of global development goals. There is a need to better understand what works to prevent violence and scale up these initiatives and sustain them over the long-term. Achieving greater gender equality requires integrating violence prevention efforts into public policies and larger systems. Breaking the intergenerational transmission of gender norms and violence perpetration is critical for reducing the prevalence of intimate partner violence. The health and well-being of women and girls, and men and boys, requires integrated approaches that tackle harmful gender norms and violence perpetration.
