# Correction: Risk Factors for Men's Lifetime Perpetration of Physical Violence against Intimate Partners: Results from the International Men and Gender Equality Survey (IMAGES) in Eight Countries

**DOI:** 10.1371/journal.pone.0126676

**Published:** 2015-05-14

**Authors:** The PLOS ONE Staff

In Table 4, the first digit is missing for each number in the 'OR' columns. Please see the corrected Table 4 here.

**Table 4:** Results from multivariate logistic regression, correlates of lifetime physical violence perpetration against a partner, presented as unadjusted odds ratios and adjusted odds ratios^a.^

**Demographic, Attitudinal, and Behavioral Variables**	**Bosnia (n = 1169)**				**Brazil (n = 617)**			
	**OR**	**95% CI**	**AOR**	**95% CI**	**OR**	**95% CI**	**AOR**	**95% CI**
*Age 18–28 (REF)*	1.00		1.00	*-*	1.00	-	1.00	* *
*Age 29–39*	1.08	0.78–1.48	1.22	0.84–1.76	1.01	0.62–1.63	1.27	0.73–2.20
*Age 40–59*	1.27	0.92–1.77	1.24	0.83–1.83	1.16	0.76–1.76	1.31	0.79–2.18
*No Schooling or Primary School (REF)*	1.00	-	1.00	*-*	1.00	-	1.00	
*Secondary School*	0.69	0.36–1.33	0.79	0.39–1.60	0.76	0.51–1.13	0.76	0.47–1.22
*Post-Secondary School*	0.57	0.29–1.13	0.72	0.34–1.55	0.26**	0.11–0.58	0.47	0.19–1.13
*Low income quartile (REF)*	1.00	-	1.00	*-*	1.00	-	1.00	
*Mid-Low Income quartile*	0.82	0.56–1.19	0.82	0.53–1.26	1.01	0.55–1.86	0.98	0.42–2.29
*Mid-High Income quartile*	0.91	0.59–1.42	1.06	0.63–1.78	0.91	0.53–1.54	1.00	0.44–2.27
*Highest Income quartile*	0.80	0.45–1.44	0.91	0.47–1.80	0.59	0.32–1.08	0.64	0.26–1.59
*Employed*	0.97	0.74–1.27	1.08	0.75–1.56	1.03	0.67–1.59	1.15	0.58–2.26
*Permissive attitudes towards VAW*	2.75**	2.03–3.72	1.34	0.93–1.95	2.40**	1.57–3.66	1.92**	1.18–3.12
*Witness of Intra-parental violence*	4.07**	2.75–6.03	2.77**	1.80–4.26	2.10**	1.32–3.33	1.71*	1.03–2.85
*GEM Score (standardized)*	0.58**	0.50–0.66	0.68**	0.58–0.80	0.81*	0.68–0.97	0.99	0.79–1.23
*Has been involved in Fights*	3.56**	2.62–4.85	2.92**	2.09–4.08	4.90**	3.26–7.37	4.04**	2.62–6.23
*Depressed*	1.50**	1.11–2.03	1.14	0.81–1.60	2.98**	1.63–5.46	2.44**	1.26–4.73

**Demographic, Attitudinal, and Behavioral Variables**	**Chile (n = 1051)**				**Croatia (n = 1152)**			
	**OR**	**95% CI**	**AOR**	**95% CI**	**OR**	**95% CI**	**AOR**	**95% CI**
*Age 18–28 (REF)*	1.00		1.00		1.00		1.00	
*Age 29–39*	1.59*	1.11–2.30	1.47	0.95–2.26	1.66**	1.20–2.31	1.66**	1.14–2.41
*Age 40–59*	2.01**	1.46–2.77	1.78**	1.18–2.69	2.86**	2.10–3.90	2.42**	1.70–3.44
*No Schooling or Primary School (REF)*	1.00	-	1.00		1.00	-	1.00	
*Secondary School*	0.58*	0.38–0.88	0.92	0.58–1.49	0.99	0.47–2.08	1.45	0.60–3.51
*Post-Secondary School*	0.51**	0.33–0.77	1.06	0.63–1.79	0.83	0.39–1.77	1.25	0.50–3.11
*Low income quartile (REF)*	1.00	-	1.00		1.00	-	1.00	
*Mid-Low Income quartile*	1.31	0.90–1.89	0.94	0.58–1.51	1.46*	1.05–2.04	1.19	0.79–1.79
*Mid-High Income quartile*	1.24	0.84–1.83	1.07	0.64–1.77	1.54*	1.07–2.22	1.35	0.86–2.13
*Highest Income quartile*	1.07	0.65–1.78	0.90	0.48–1.69	1.09	0.74–1.60	0.98	0.60–1.60
*Employed*	1.57**	1.15–2.15	1.44	0.95–2.20	1.21	0.91–1.60	1.03	0.72–1.48
*Permissive attitudes towards VAW*	2.59**	1.73–3.89	1.90**	1.18–3.04	4.50**	3.05–6.63	3.14**	2.00–4.95
*Witness of Intra-parental violence*	3.20**	2.42–4.23	2.68**	1.99–3.60	2.97**	2.14–4.11	2.10**	1.47–3.00
*GEM Score (standardized)*	0.74**	0.65–0.84	0.87	0.74–1.01	0.68**	0.60–0.77	0.87	0.75–1.02
*Has been involved in Fights*	2.75**	1.96–3.87	2.91**	1.98–4.27	2.43**	1.79–3.29	1.98**	1.41–2.76
*Depressed*	1.64**	1.17–2.31	1.50*	1.02–2.21	1.55**	1.20–2.02	1.29	0.97–1.72

**Demographic, Attitudinal, and Behavioral Variables**	**DRC (n = 539)**				**India (n = 917)**			
	**OR**	**95% CI**	**AOR**	**95% CI**	**OR**	**95% CI**	**AOR**	**95% CI**
*Age 18–28 (REF)*	1.00		1.00	*-*	1.00	-	1.00	
*Age 29–39*	2.25**	1.36–3.70	2.47**	1.45–4.22	1.80**	1.22–2.65	1.93**	1.26–2.95
*Age 40–59*	3.15**	1.92–5.17	3.91**	2.29–6.69	2.31**	1.55–3.44	2.57**	1.65–3.99
*No Schooling or Primary School (REF)*	1.00	-	1.00	*-*	1.00	-	1.00	
*Secondary School*	1.71**	1.17–2.51	1.90**	1.26–2.87	0.70*	0.50–0.99	0.68	0.46–1.01
*Post-Secondary School*	1.40	0.87–2.24	2.02*	1.14–3.56	0.49**	0.35–0.69	0.47**	0.30–0.74
*Low income quartile (REF)*	1.00	-	1.00	*-*	1.00	-	1.00	
*Mid-Low Income quartile*	2.98**	1.51–5.87	2.80**	1.36–5.79	1.20	0.84–1.73	1.42	0.94–2.15
*Mid-High Income quartile*	1.80	0.91–3.57	1.17	0.56–2.47	0.98	0.67–1.43	1.34	0.84–2.14
*Highest Income quartile*	1.17	0.45–3.01	0.76	0.26–2.17	0.91	0.62–1.33	1.35	0.82–2.24
*Employed*	1.32	0.92–1.90	1.10	0.71–1.71	1.06	0.55–2.08	1.41	0.65–3.05
*Permissive attitudes towards VAW*	1.86**	1.31–2.66	1.52	0.99–2.34	1.47**	1.11–1.96	1.28	0.89–1.84
*Witness of Intra-parental violence*	1.26	0.90–1.78	1.26	0.86–1.83	4.09**	3.08–5.42	3.73**	2.77–5.04
*GEM Score (standardized)*	0.80**	0.67–0.95	0.92	0.75–1.14	0.88	0.77–1.02	1.03	0.85–1.24
*Has been involved in Fights*	1.17	0.70–1.96	1.32	0.75–2.33	3.39**	1.96–5.88	3.51**	1.92–6.43
*Depressed*	NA	NA—NA	NA	NA	1.67**	1.24–2.25	1.65**	1.18–2.32

**Demographic, Attitudinal, and Behavioral Variables**	**Mexico (n = 895)**				**Rwanda (n = 1456)**			
	**OR**	**95% CI**	**AOR**	**95% CI**	**OR**	**95% CI**	**AOR**	**95% CI**
*Age 18–28 (REF)*	1.00		1.00		1.00		1.00	
*Age 29–39*	1.52	0.96–2.40	1.29	0.78–2.16	1.40	0.98–2.01	1.43	0.99–2.08
*Age 40–59*	1.54*	1.01–2.35	1.18	0.72–1.93	1.46*	1.03–2.07	1.55*	1.08–2.22
*No Schooling or Primary School (REF)*	1.00	-	1.00		1.00	-	1.00	
*Secondary School*	1.27	0.71–2.26	1.48	0.77–2.81	1.18	0.85–1.63	1.04	0.73–1.48
*Post-Secondary School*	0.82	0.48–1.39	1.40	0.75–2.61	0.71	0.41–1.23	0.68	0.38–1.21
*Low income quartile (REF)*	1.00	-	1.00		1.00	-	1.00	
*Mid-Low Income quartile*	0.86	0.45–1.66	0.95	0.47–1.93	0.95	0.71–1.28	1.01	0.74–1.37
*Mid-High Income quartile*	1.29	0.69–2.39	1.64	0.83–3.23	1.15	0.85–1.54	1.17	0.86–1.59
*Highest Income quartile*	0.87	0.46–1.64	1.19	0.59–2.42	1.08	0.79–1.49	0.92	0.76–1.49
*Employed*	1.70*	1.04–2.78	1.87	0.98–3.54	1.02	0.49–2.11	0.82	0.35–1.94
*Permissive attitudes towards VAW*	4.55**	2.54–8.15	2.55**	1.31–4.97	1.55**	1.19–2.02	1.33	0.99–1.80
*Witness of Intra-parental violence*	3.81**	2.58–5.63	3.08**	2.00–4.72	2.42**	1.95–3.00	2.34**	1.88–2.92
*GEM Score (standardized)*	0.61**	0.52–0.72	0.68**	0.56–0.82	0.88*	0.79–0.98	0.94	0.83–1.06
*Has been involved in Fights*	2.67**	1.69–4.21	2.19**	1.31–3.66	1.69*	1.06–2.70	1.67*	1.02–2.73
*Depressed*	2.42**	1.45–4.01	1.85*	1.06–3.23	NA	NA	NA	NA

* p<.05,

**p<.01; CI = Confidence Intervals, OR = unadjusted odds ratio; AOR = Odds ratios adjusted for other variables in table^; a^Adjusted for all other variables presented in table
